# Thermal Property Analysis of Axle Load Sensors for Weighing Vehicles in Weigh-in-Motion System

**DOI:** 10.3390/s16122143

**Published:** 2016-12-15

**Authors:** Piotr Burnos, Janusz Gajda

**Affiliations:** Department of Measurement and Electronics, AGH University of Science and Technology, 30-059 Krakow, Poland; jgajda@agh.edu.pl

**Keywords:** vehicle axle load sensors, Weigh-in-Motion, pavement temperature

## Abstract

Systems which permit the weighing of vehicles in motion are called dynamic Weigh-in-Motion scales. In such systems, axle load sensors are embedded in the pavement. Among the influencing factors that negatively affect weighing accuracy is the pavement temperature. This paper presents a detailed analysis of this phenomenon and describes the properties of polymer, quartz and bending plate load sensors. The studies were conducted in two ways: at roadside Weigh-in-Motion sites and at a laboratory using a climate chamber. For accuracy assessment of roadside systems, the reference vehicle method was used. The pavement temperature influence on the weighing error was experimentally investigated as well as a non-uniform temperature distribution along and across the Weigh-in-Motion site. Tests carried out in the climatic chamber allowed the influence of temperature on the sensor intrinsic error to be determined. The results presented clearly show that all kinds of sensors are temperature sensitive. This is a new finding, as up to now the quartz and bending plate sensors were considered insensitive to this factor.

## 1. Introduction

In order to protect roads and bridges against overloaded vehicles, numerous and varied steps have been taken. One of these steps, though not necessarily an effective one, is static mass and axle load enforcement. This type of enforcement involves stopping random vehicles and directing them to a weighing station, where the vehicles are weighed on precise (with a relative error of 1%) static scales. An alternative is to weigh vehicles directly on the lane that they are traveling on, without the need to stop them.

**Figure 1:**
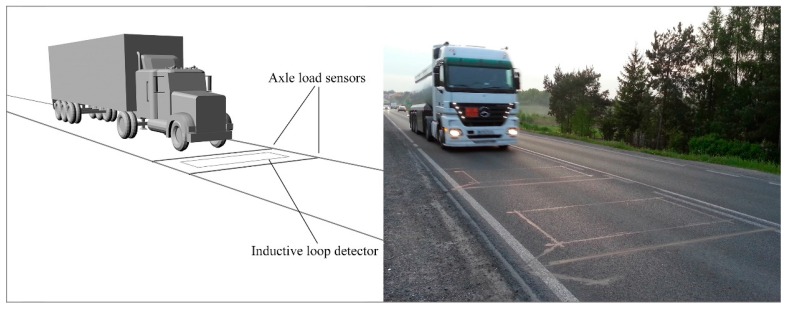
Diagram and photo of a WIM station.

Systems which allow the weighing of vehicles in motion are called dynamic Weigh-in-Motion (WIM) scales. In such systems, wheel or axle load sensors are embedded in the pavement perpendicular to the traffic direction. Within this group are distinguished Low Speed WIM, High Speed WIM and Multi-Sensor WIM systems. Each of them can be equipped with various types of load sensors, such as polymer, quartz, bending plate, capacitive or fiber-optic [1,2,3]. The number of load sensors used and their type depends on the desired accuracy of the weighing results. The common systems consist of two load sensors, and are referred to as High Speed WIM (HS-WIM). The dynamics of a moving vehicle and the impact of the other disturbing quantities mean that weighing errors in HS-WIM systems reach as much as 15% for axle load and 5% for gross vehicle weight. For this reason, such systems are not used by authorities for direct mass enforcement and punishment of carriers using overloaded vehicles. Currently, such systems are used only for preselection of vehicles suspected of being overloaded, which are next directed to static scale weighing stations. Figure 1 shows a diagram and a photo of a WIM system. 

**Figure 2:**
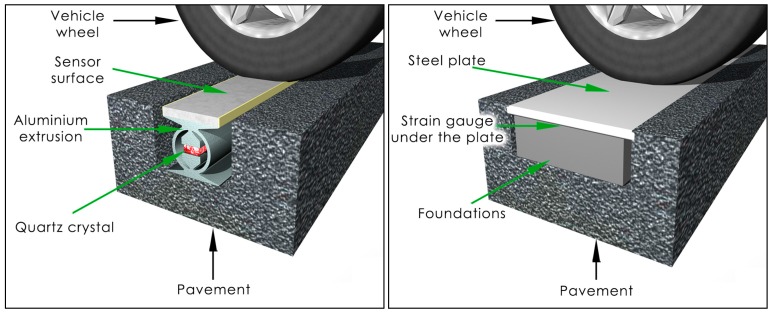
Quartz and bending plate sensors embedded in the pavement.

Nowadays, load sensors made with three different technologies are most frequently used: polymer, quartz and bending plate [3]. In each case, the sensors are embedded in the road pavement. The pavement plays a role in transferring the force of the vehicle’s wheel to the sensor. Thus, the fragment of the pavement in which the sensor is installed becomes a part of the measuring system, bringing with it a host of negative consequences. This means that the variety of disturbing quantities affecting the mechanical properties of the pavement and sensors are difficult to study and describe. Figure 2 shows the way that sensors are embedded in the pavement.

In many countries extensive research has been conducted over a number of years into the application of WIM systems for direct enforcement purposes but without success [4,5]. The fundamental problems, which so far have not been solved, involve ensuring high and constant accuracy of WIM systems and the quantitative assessment of factors disturbing the weighing process. The need for WIM enforcement systems implementation has also been discerned in EU countries, as is confirmed by initial reports appearing in international journals [6,7,8].

From the theory of bitumic pavements it is known that the one of the reasons for low weighing accuracy in WIM systems is the phenomena which occur in the pavement/load sensor complex, under the impact of changes in pavement temperature.

The phenomena which occur at the juncture of the pavement/sensor complex have not yet been studied or explained. Without explanation of these phenomena, it is not possible to build a WIM system with accuracy comparable to that of the static scales. Achieving such accuracy will enable the use of WIM systems for direct elimination of overloaded vehicles from the road.

The goal of this paper is to achieve progress on closing the gap within assessment of the properties of polymer, quartz and bending plate load sensors for WIM systems. The studies were conducted in two ways: at roadside WIM sites equipped with wheel/axle load sensors built with various technologies, and at a laboratory using a climate chamber. We present a quantitative assessment of the influence of pavement temperature on the vehicle weighing error caused by the intrinsic properties of the load sensors and the thermal properties of the pavement. In particular, the long-term accuracy assessment of WIM systems equipped with all kinds of sensors has been shown. The characteristics describing the change in system error as a function of temperature have been experimentally determined. We also present the effects of the temperature gradient occurring on a multi-sensor site. 

In the final part of this paper we will demonstrate that currently adopted recommendations from COST 323 [9] concerning temperature stability during system calibration and when testing its accuracy are not correct and need adjustments.

### Literature Review

Ongoing research on WIM systems is aimed at their application for enforcement purposes. Practical implementation of this idea is subject to two conditions: defining detailed procedures for legalization and metrological control of WIM systems, and defining technical requirements for such WIM systems. The second problem ensues from the high sensitivity of WIM systems to weather conditions, environmental influences and factors directly associated with the weighed vehicle and its behavior at the WIM site [10]. The two main factors with the most significant effect on the WIM system’s accuracy are the pavement temperature changes and vehicle speed.

At the beginning of the 21st century, Papagiannakis, Johnston and Alavi carried out studies on piezoelectric sensors. In one such study [11], they focused on laboratory evaluation of the fatigue characteristics of piezoelectric WIM sensors under wet and dry conditions. In another paper written by the same authors [12], the results of the field evaluation of WIM sensors of two manufacturers, namely Vibracoax (VC) and Measurements Specialties Incorporated (MSI), were presented. In these experiments the influence of the pavement temperature on the sensors’ output signal was investigated. Studies have shown that the level of the raw signal depends on the temperature sensors installed in the asphalt concrete pavement. Concurrent with the increasing pavement temperature, the signal level of the MSI sensors increased, while it decreased for the VC sensors. 

Temperature properties of WIM sensors are also addressed in [13]. The paper describes the laboratory and field tests of the RoadTrax BL sensor manufactured by MSI. During tests, all the examined sensors exhibited an increase in the signal level with the increased temperature of the asphalt concrete pavement. The coefficient in this relationship expressed in volts per degree Celsius is as high as 0.162.

The influence of the pavement temperature and vehicle speed on the weighing error in WIM systems employing piezoelectric polymer sensors is also emphasised in the paper [14]. The results presented confirm that polymer sensors and ceramic sensors are considered to be temperature sensitive, while quartz sensors are insensitive to temperature. The comparison of sensors in terms of sensitivity to changes in vehicle speed resulted in the conclusion that the polymer sensors are the least sensitive to speed change, the quartz sensors have better accuracy at higher speed, and the results for the ceramic sensors are more scattered than for the other two types of sensors overall.

A similar conclusion is reached by Vaziri in his PhD thesis [15] and further publications [16]. The aim of that research was to explore the behavior of WIM piezoelectric sensors under different environmental conditions and vehicle behavior during weighing. The author conducted field tests on two WIM sites involving three types of load sensors: quartz (Kistler), polymer piezoelectric (MSI) and ceramic piezoelectric (Electronique Controle Mesure—ECM). Unfortunately, the reliability of the presented results seems to be very limited. The author measured the temperature of the air instead of the pavement and only within a small range (8–16 °C), which is insufficient. With respect to the effect of the vehicle speed on the performance of the WIM sensors, the same objection may be formulated: test speeds were chosen for 30, 50 and 70 km/h. It seems to not be representative for heavy vehicles, as the average speed on national roads and highways, depending on the vehicle class, is within the range of 60–110 km/h.

The properties of WIM sites were also examined by the authors of this paper. Since 2005, a series of test has been performed at a Multi-Sensor WIM (MS-WIM) site equipped with 16 polymer axle load sensors provided by MSI [17]. Long-term tests clearly showed a strong relationship between the pavement temperature and weighing results. A model of this relationship was first introduced by Burnos in 2008 [18] and further investigated in [19].

From the data obtained during all the aforementioned works, it was not possible to determine if the increase in the sensor’s output signal is caused by a decrease in pavement stiffness or by the properties of the sensor itself. Such a study for polymer sensors was undertaken by the authors and described in [20] where the results of tests in a climatic chamber, as well as of long-term tests at the WIM site, were presented. Quantitative assessments of the temperature impact on the sensor itself and on the asphalt concrete pavement stiffness were described.

Almost all the mentioned works exclusively concern the thermal properties of polymer sensors. Fields that still need more investigation are the influence of the vehicle speed on the weighing results and the thermal properties of quartz and bending plate sensors. For instance, Kistler gives a temperature coefficient of sensitivity of −0.02%/°C [21]. This value, however, refers solely to the piezoelectric material used, and does not take into account temperature effects occurring after the sensor installation in the pavement. This paper fills this gap in the knowledge.

## 2. Methodology and Experiment Setup

Regarding pavement/load sensor complexes, two kinds of weighing errors are distinguished:

- Sensor intrinsic error: the source of this error is a change in the sensor’s electrical parameters under the impact of the change of the temperature.
- Pavement/sensor complex external error: the superposition of the sensor intrinsic error and errors which occur after sensor installation in the road pavement. The source of the additional errors in this case is the pavement, as its properties depend on the temperature and duration of force applied by the vehicle wheel on the pavement/sensor complex.

Taking into account the two kinds of errors present in the pavement/load sensor complex, the studies were conducted in two ways: at roadside WIM sites equipped with wheel/axle load sensors built with various technologies, and at a laboratory using a climate chamber. These two methods differ in terms of the required infrastructure, the duration of the measurements, the kind of experiments that can be carried out, and cost. However, they are complementary. Tests at the climatic chamber allowed the authors to determine the influence of the temperature on the sensor intrinsic error, and tests at the roadside WIM site permitted the evaluation of the pavement/load sensor external error.

The characteristics that describe the roadside WIM system properties have been determined using the reference vehicles method [18]. The basis of the method is the assumption that results of weighing a certain class of vehicles participating in traffic can be used as the reference value. In Poland, five-axle articulated vehicles, including two-axle tractors and three-axle semi-trailers, are categorized into this class. The load of the first axle of these vehicles is most weakly correlated with the loads of the other axles and the gross vehicle weight compared to the other vehicle classes. These features mean that the load of the first axle of the reference vehicle is stable, and to a small degree it depends on the carried load. As a consequence, as a reference for the evaluation of the WIM system’s accuracy, the mean value of the first axle load of the reference vehicles was chosen. In a population of two-axle tractors with three-axle semi-trailers, the mean value of the first axle load equals 61.670 N. Reference vehicles are regular traffic participants and can be easily recognized by the WIM system, because the distance between the three axles of the semi-trailer is normalized and equals 1.3 m. The population of such vehicles is usually large in comparison to all other heavy vehicle classes. All these features of reference vehicles allow for long-term tests at the roadside WIM site, and evaluation of the temperature properties of the load sensors (measurements taken in different temperatures over the year). Tests carried out at the roadside WIM site allowed the authors to determine the pavement/load sensor external error. Multiple repetitions of this experiment at different temperatures make it possible to define characteristics which give a quantitative description of the influence of disturbing quantities on the WIM system error.

The accuracy assessment of the load sensors consists of analysis of the weighing results of the reference vehicles’ first axle at the specified pavement temperature and vehicle speeds. The relative error of weighing results *δ* is computed from Equation (1).

(1) $$\delta =\frac{1}{N}\sum_{i=1}^{N}\frac{Ldyn_{i}-Lstat}{Lstat},$$

where:

*Ldyn_i_*—*i*-th axle weighing results of the moving reference vehicle obtained from the WIM system
*Lstat*—reference value of axle load, in this case the mean value of the first axle load of the reference vehicles (61.670 N)
*I* = 1, 2, …, *N*—number of weighing results of the first axle of the reference vehicles, taken at the specific value of the temperature and the vehicle’ speed

Averaging in Equation (1) is over the set of weighing results taken at a given pavement temperature and vehicle speed. This is necessary because of deviation of the first axle load of the reference vehicles, the vertical bouncing of the vehicle, and other errors that may occur in the WIM system. For each pavement temperature or vehicle speed, at least several dozens of weighing results are taken to calculate the relative error.

Regarding laboratory tests, a CTS C-40/100 climatic chamber was used. Model C-40 allows temperature to be regulated from −40 °C to +180 °C. The chamber test space dimension is 0.5 × 0.5 × 0.4 m. Tests carried out in the climatic chamber allowed the authors to determine the influence of temperature on the sensor intrinsic error. No load was applied on the sensor. The measurements were made for two polymer sensors of the same type. In the first case only the sensor was placed in the chamber, whereas in the second case the sensor with the cable connecting it to the measuring system was included.

## 3. Results

To evaluate the thermal properties of the WIM systems equipped with different types of sensors, data from three roadside WIM sites located in Poland were analyzed:

- Polymer load sensor Roadtrax^®^ Brass Linguini^®^—made by TE Connectivity Ltd. [22] (former Measurement Specialties, Inc., Schaffhausen, Switzerland)—site in the city of Gardawice on national road No. 81
- Quartz sensor Lineas^®^ made by Kistler (Winterthur, Switzerland) [21]—site in the city of Rudawa on national road No. 79
- Bending plate sensor PAT DAW 100^®^ made by International Road Dynamics^®^ (Saskatoon, Canada)—site in the city of Zabow on national road No. 2

The data were collected in different periods depending on the weighing site, but the operating time of each site was not shorter than six months. The aim of the data collection was to record the weighing results of reference vehicles. Comparing the weighing result of the first axle of these vehicles from the WIM system to the mean value of the first axle load allowed the evaluation of the system’s accuracy. In total, over four million vehicles of all classes were recorded, including cars, vans, buses, etc., of which 0.2 million were records of reference vehicles: 75,000 at the site with polymer sensors, 67,000 at the site with quartz sensors and 72,000 for at the site with bending plate sensors.

### 3.1. Pavement/Load Sensor External Errors Due to Temperature Influence

**Figure 3:**
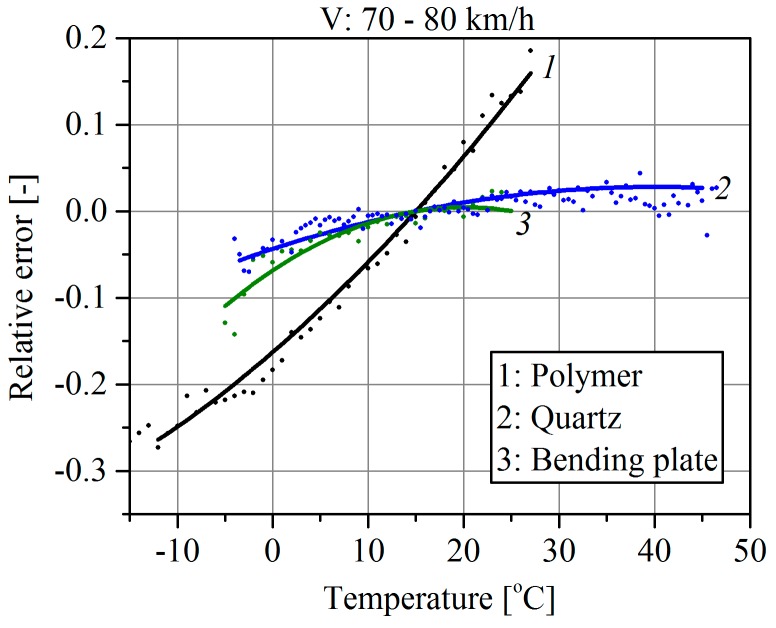
Comparison of temperature characteristics of polymer, quartz and bending plate sensors installed in pavement.

The analysis of the results of the reference vehicles’ first axle weighing consists of assessment of the error value (1) at the specified pavement temperature. To limit the impact of changes in the vehicle speed on the results, the measurements were limited to the range of the reference vehicles’ speeds of 70–80 km/h. Characteristics shown in Figure 3 are a second^-^order polynomial approximation (the best fit) of the relative error (1). Each approximated point is the average value of the results of weighing at least several dozen vehicles. It should be emphasized that these characteristics take into account the temperature influence on the intrinsic properties of the sensor and on the pavement parameters, and hence the external relative error of vehicle weighing. All WIM systems were calibrated at a temperature of 15 °C.

From the characteristics shown in Figure 3, the following conclusions can be formulated:

- WIM systems employing polymer sensors were definitely more sensitive to temperature changes than quartz and bending plate sensors. For this type of sensor, a temperature change within the range −10 °C to +30 °C produced, as an effect, a change in the weighing error of approximately 50%. In the case of quartz sensors and bending plates, that change was approximately 7%.
- Considering the daily variation of the pavement temperature, it was not possible to achieve better accuracy than 7% in a WIM system equipped with quartz or bending plate sensors without temperature correction algorithms.

The knowledge of the polynomial models enables online temperature correction of the weighing error but it requires continuous measuring of the pavement temperature at a depth similar to that at which the load sensors are installed.

### 3.2. Influence of a Non-Uniform Distribution of Pavement Temperature along the Site on Weighing Results

**Figure 4:**
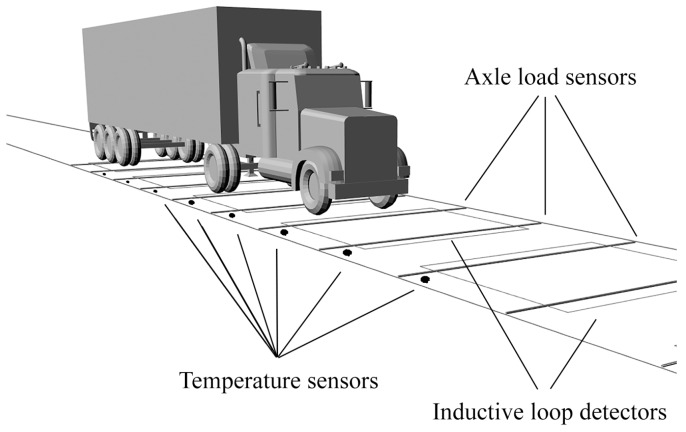
A view of the Multi-Sensor WIM site with temperature sensors installed along the road.

In the case of Multi-Sensor WIMs, the length of the site may exceed 10 m. In such a long measuring system, the pavement temperature distribution along the site, in general, is non-uniform. This yields the necessity of temperature sensor installation along the site. Figure 4 shows the Multi-Sensor WIM site equipped with temperature sensors.

**Figure 5:**
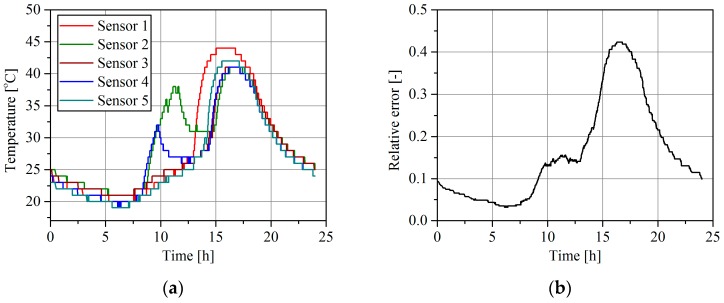
(**a**) Daily pavement temperature change at the MS-WIM site at five measurement points, distributed along the site; (**b**) A sample daily variation of the weighing error (1) at the MS-WIM site due to the pavement temperature change.

Figure 5a shows sample results of the daily change of the pavement temperature at the MS-WIM site equipped with five temperature sensors distributed along the site, and 16 polymer load sensors. The weighing result in the MS-WIM system is determined as the arithmetic mean value of the measurements obtained from the successive load sensors. The error presented in Figure 5b is therefore a total weighing error and it consists of errors contributed by all 16 load sensors. The characteristic presented in Figure 5b describes the case where the temperature correction was not used.

As can be seen from the characteristics in Figure 5a, the asphalt temperature changed during the day by as much as 25 °C. This may be considered a typical range in the summer season in Poland. It can also be noticed that the temperature distribution along the site was not uniform. The length of MS-WIM sites depends on the number of load sensors installed and may exceed 10 m. Therefore, temperatures at different points of a site may differ significantly, chiefly due to the non-uniform insolation caused by neighboring trees and high buildings situated along the road. On HS-WIM sites equipped with only two sensors, the distance between sensors is limited to 1–2 m, thus effects produced by the non-uniform temperature distribution along the WIM site are much smaller.

### 3.3. Influence of a Non-Uniform Distribution of Pavement Temperature across the Site on Weighing Results

**Figure 6:**
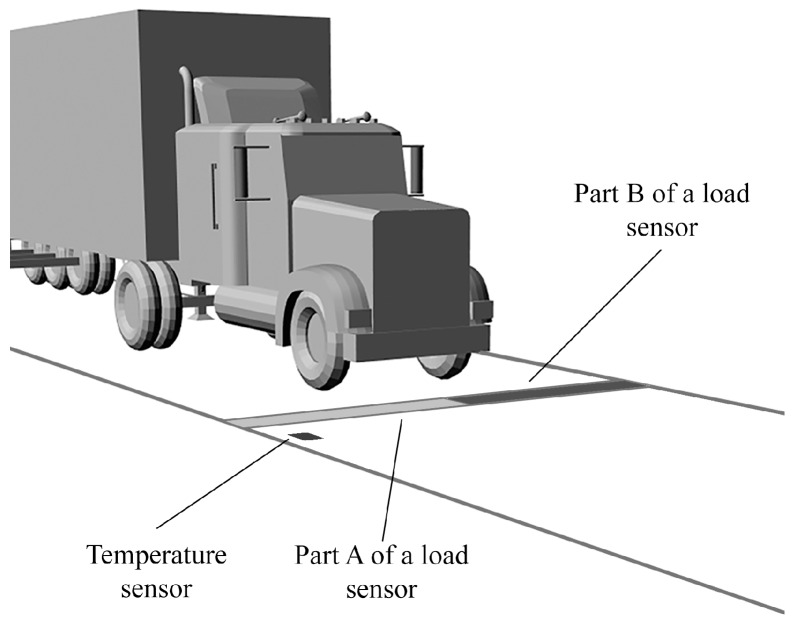
A view of two parts of the load sensor at two different temperatures.

A non-uniform temperature distribution is observed not only along the site but also across it, i.e., along each load sensor. In this case also it may be caused by shadows cast onto the pavement by roadside objects. That means two parts of a load sensor may be exposed to different temperatures. Let us assume the situation shown in Figure 6. Two parts of the load sensor (part A and part B) are operated at different temperatures, but only the temperature of part A is measured. 

**Figure 7:**
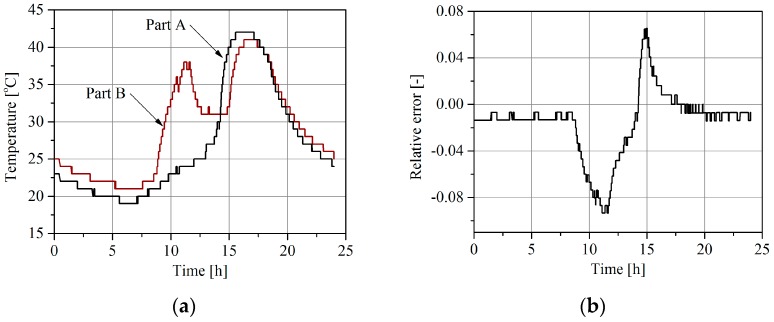
An example of daily temperature variations in two parts of a load sensor: (**a**) Part A—temperature of part A, Part B—temperature of part B; (**b**) relative weighing error due to different temperatures of parts A and B.

Additionally, let us assume that temperature correction of the weighing result based on the polynomial model (Figure 3) is implemented in the system. If the temperature correction algorithm uses the temperature measurement of part A it means that such a compensation is not correct because part B of the load sensor has a different temperature. Daily changes in temperature of both parts of the load sensor, observable in the summer season, are shown in Figure 7a. Assuming that the temperature correction is based on the measurement result of the temperature of part A, the relative weighing error due to the different temperatures of both parts of the load sensor changes during a day is shown in Figure 7b. This is one of the total weighing error components that is caused by temperature influence.

### 3.4. Sensor Intrinsic Errors 

**Figure 8:**
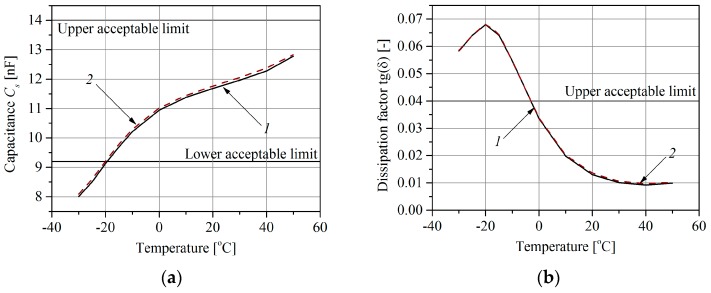
The influence of temperature on: (**a**) the equivalent capacitance *C_s_* of a piezoelectric polymer sensor; (**b**) dissipation factor *tg*(*δ*), for 1: sensor with connecting cable, 2: sensor without connecting cable.

The objective of the tests carried out in a climatic chamber was to determine the influence of the temperature on changes of the polymer sensor equivalent parameters of capacitance *C_s_* and dissipation factor *tg*(*δ*). The research has been described in detail in [19,20]. Only polymer sensors were tested because the sizes of the other sensors exceed the dimensions of the climate chamber. Two cases were considered. In the first case only the sensor was placed in the chamber, whereas in the second case the sensor was placed with the cable connecting it to the measuring system. The measurement results of the sensor equivalent parameters are shown in Figure 8. The measurements were performed over a temperature range of −30 °C to +50 °C, according to the requirements of the standard [23]. 

**Figure 9:**
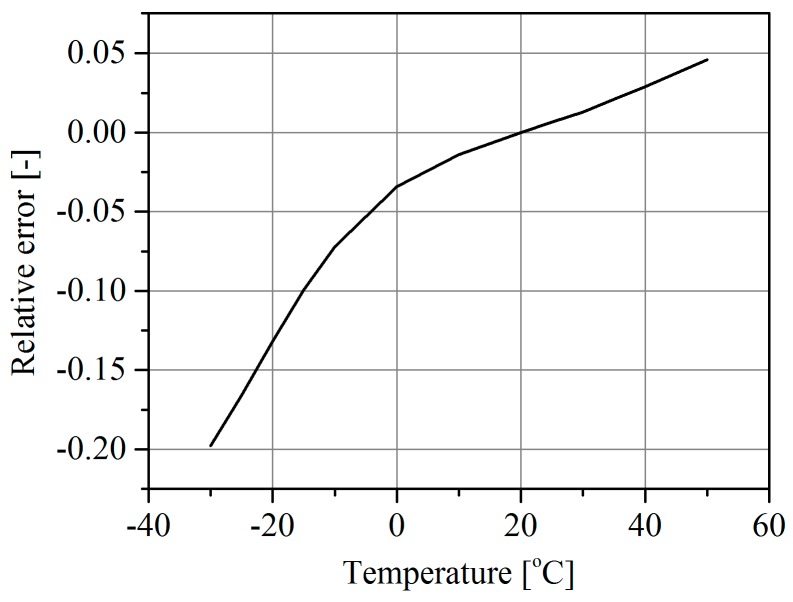
The weighing error in a WIM system equipped with piezoelectric polymer sensors, arising exclusively from a change in the sensor parameters due to a change in temperature (intrinsic error).

The weighing intrinsic error arises from the difference between the actual sensor temperature value and the reference temperature value at which the WIM system was calibrated (in this test the reference temperature was +20 °C). The relative value of the error is computed from Equation (1). The plot of error (1) versus the sensor temperature is shown in Figure 9. As follows from this characteristic, a rise in temperature of 20 °C over the reference temperature results in a weighing error of about 4%. At extremely low temperatures this error may be contained within the range 10%–20%.

## 4. Conclusions and Future Work

Achieving a high and known accuracy of WIM systems will enable their use for direct enforcement purposes. Due to the influence of numerous factors limiting this accuracy, this is not an easy task.

The shape of the characteristic in Figure 3, and at the same time the sensitivity of the weighing results to temperature changes, depends on the pavement mechanical properties and varies from site to site. An explanation of system behavior would require to take a stiffness model of the pavement into account. This model is complex and nonlinear in nature with three variables: the stiffness coefficient, vehicle speed and axle load. This will be the aim of further studies in cooperation with the Gdansk University of Technology.

The unequal temperature distribution along and across the WIM site may also be the cause of significant weighing errors. In order to limit the influence of this factor, it is essential to install at least two temperature sensors located at the ends of each load sensor. Locations in which this unequal distribution is highly likely (such as densely urbanized areas, areas where tree shadows influence pavement temperature, or road infrastructure objects) should also be avoided as WIM sites.

Finally, comparing the characteristics determined for polymer sensors shown in Figure 3 and Figure 9 allowed two causes of the weighing errors to be separated quantitatively. The influence of the sensor intrinsic error was −12% to +2%, and the influence of the pavement parameter change (sensor external error) was −30% to +20% over a temperature change range of −20 °C to +30 °C. The intrinsic sensor error caused by the temperature change is dependent on the technology and materials used in its manufacture.

In the authors’ opinion, the pavement temperature is a significant influencing factor; moreover, its value varies over a wide range in relatively short time intervals. This phenomenon concerns all kinds of sensors: polymer, quartz and bending plate load sensors. The results presented show clearly that in the case of the Lineas load sensors manufactured by Kistler, their temperature sensitivity, after installation in the pavement, was about 7% within the temperature range of −10 °C to +30 °C. It is therefore necessary to reconsider views about the method of WIM system calibration and assessing their accuracy using the pre-weighed vehicle method proposed in COST 323 [9]. In the future, accurate Multi-Sensor WIM systems intended for mass enforcement should be calibrated and tested over a wide range of temperature changes similarly as they are tested over a wide range of pre-weighed vehicle speeds. Furthermore, as a result of model and laboratory research and on-site experiments, it has been demonstrated that in order to achieve a high accuracy level, the WIM site should be equipped with a multi-point temperature measuring system. 

The contribution of this work is aimed at developing a Polish WIM system for direct overloading enforcement. To achieve this goal, in 2014 the authors initiated a group within the Intelligent Transportation Systems Cluster (ITS Cluster), which brought together Polish universities, research institutes and commercial companies.
